# Geo-Relationship between Cancer Cases and the Environment by GIS: A Case Study of Trabzon in Turkey

**DOI:** 10.3390/ijerph6123190

**Published:** 2009-12-11

**Authors:** Tahsin Yomralioglu, Ebru H. Colak, Arif C. Aydinoglu

**Affiliations:** 1 Department of Geomatics Engineering, Istanbul Technical University, 34469 Maslak, Istanbul, Turkey; E-Mail: aaydinoglu@itu.edu.tr; 2 Department of Geomatics Engineering, Karadeniz Technical University, GISLab, 61080, Trabzon, Turkey; E-Mail: ecolak@ktu.edu.tr

**Keywords:** GIS, cancer, epidemiology, statistical map, cancer map

## Abstract

Cancer is an important health issue in Turkey because it ranks as the second cause of death in the country. Examination of the relationships between the distribution of cancer cases and geo-environmental factors is significant in determining the causes of cancer. In this study, GIS were used to provide data about the distribution of cancer types in Trabzon province, Turkey. To determine the cancer occurrence density, the cancer incidence rates were calculated according to local census data, then a cancer density map was produced, and correlations between cancer types and geographical factors were examined.

## 1. Introduction

Cancer is a major health issue and one of the most common diseases in the World, with more than 11 million cases being diagnosed every year [1]. The World Health Organization (WHO) estimated that of 58 million lives lost globally in 2005, 7.6 million died from cancer and 84 million people will die over the next 10 years if no action is taken. More than 70% of all cancer related deaths occur in countries where the population has a medium to low level standard of living and limited resources for the prevention, diagnosis, and treatment of cancer [2].

In Turkey, cancer has become the second major cause of death [3]. In 2005, approximately 52,000 people died from cancer in Turkey and 37,000 were under the age of 70 [4]. According to Cancer Research UK (2002) the average cancer incidence rates are 330.5 in North America, 266.7 in Western Europe and 195.9 in Eastern Europe. In the region where Turkey is located the average incidence rate is 130.2 per 100,000 population [5]. Cancer not only has a high level of mortality but also can result in serious disability and impacts on the productivity of the labour force. Treatment costs are high and have major implications for government expenditures on health services.

The Cancer Control Program (CCP) initiated by the WHO aims to reduce the incidence of cancer and cancer related deaths, and to improve the quality of life of cancer patients [6]. Within the context of this program, descriptive statistics presenting the distribution of cancer cases, existing density of cancer cases, and the most common cancer types in the country should be determined [7]. In order to enforce cancer control strategies effectively in Turkey, firstly, statistical data about cancer cases should be collected. Then, cancer maps need to be created to obtain a realistic analysis of the spatial distribution of the cases. These maps facilitate the determination of the regions with greatest density of cancer cases and the spatial examination of the environmental factors possibly causing cancer in these regions [8]. In this context, Geographic Information Systems (GIS) can be used to map and analyze the geographical distribution of populations at risk, to determine environmental risk factors, to explore associations between risk factors and health outcomes, and to address health issues [9,10].

This research aimed to examine the relationship between the distribution of cancer cases and environmental factors in Trabzon province, Turkey. Firstly, various data collected with GIS & Remote Sensing (RS) techniques are combined in a spatial database. Then, the geo-statistical maps displaying the cancer density within administrative units were produced with regard to the cancer cases of Trabzon province for the year 2004. Thus, the distribution of the cancer cases was investigated in conjunction with population and environmental factors. The distribution map of cancer cases based on land cover was produced to examine whether there is any statistical relationship between land cover and cancer types.

## 2. Method

### 2.1. Background: Cancer Registry Process in Turkey

Disease Centers collect information about notable diseases In Turkey and maintain disease registries on databases [11]. Cancer case data is collected by local health centers using a recording system protected by laws governing privacy and confidentiality. This data includes the residential address of the cancer patients. In this way, with analytical studies the trends of cancer incidence and mortality in different populations can spatially be determined in order to examine the relationships between incidence, mortality, and environmental risk factors [12].

By supporting an epidemiological study cancer registries play an important role in investigating the relationship between the causes and frequency trends of cancer. The main characteristics of cancer registries are to monitor trends in cancer incidence, prevalence, and mortality over time and between different areas and social groups. These depend on the implementation of the Cancer Control Program and the available cancer registries [13]. In order to put a cancer control program into practice, reliable population-based information on cancer incidence, prevalence, and mortality rates is required.

### 2.2. Geographical Information Systems and Cancer Maps

The earliest disease maps were produced in Germany over two hundred years ago. Then in 1855, John Snow’s dot maps of a cholera epidemic in UK were the first published and became the most famous example of spatial epidemiology [14]. The first cancer maps in colour were produced in 1875 [15]. In the 1970s, the use of GIS increased among epidemiologists and public health researchers in conjunction with the development of new technologies [16]. The recent advances and increased awareness of GIS and mapping techniques have created new opportunities for public health administrators to enhance their planning, analysis, and monitoring capabilities. Thus, they are able to assess the relationship between public health and the geographic characteristics of residential area [17–19]. Specifically, in terms of epidemiologic studies for cancer, GIS supports the exploration of the relationship between environmental risk factors and disease. GIS is widely utilized in order to produce cancer maps and to implement statistical and spatial analysis for cancer globally [20–28]. There are a large number of statistical or geo-statistical methods for cancer investigation in epidemiology [29–31].

### 2.3. The Registration Process of Cancer Cases in Turkey

In Turkey, cancer registries have been collected since 1982, the registration process being conducted by the Cancer Control Department of the Turkish Ministry of Health. In the past, cancer cases were registered in a disorganized manner without complying with any national or international standard. Therefore, to determine the overall profile of the incidence of cancer in Turkey the “Turkish Cancer Registry and Incidence” project was initiated in 1992 to monitor cancer cases countrywide. Cancer Registry Centers (CRCs) were founded in 11 provinces and the cancer cases began to be recorded according to the International Classification of Diseases (ICD-9) coding system. This standard is used to develop a reliable database for cancer cases in these centers [32].

Since 2004, the Trabzon CRC, a subdivision of Provincial Health Directorate of Trabzon, has been collecting active cancer data from all health institutions and hospitals in Trabzon province in accordance with ICD-9 standard. This center collaborates with the Cancer Division of Ministry of Health and coordinates the activities of all the allied units. The personnel of this center enter the data into computers, control the quality of all the data, prevent duplications, evaluate the data, and prepare reports explaining the cancer incidence rates.

### 2.4. The Study Area

**Figure 1.**
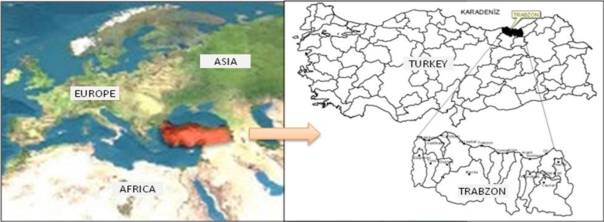
Study Area.

Trabzon, the chosen study area, is situated between 38°30′–40°30′ east longitude and 40°30′–41°30′ north latitude, with the Black Sea to the north, Gumushane and Bayburt on the southern side, Rize on the eastern side, and Giresun on the western side. Trabzon province has a socio-economic development ranking of 38th out of the 81 provinces of Turkey [33]. As can be seen on the map (Figure 1), Trabzon province comprises 17 county towns and 537 villages and covers an area of 4,664 km^2^. According to 2008 census conducted by the Turkey Statistics Institute the population of Trabzon was 748,982, with a density of 161 people per square kilometre [34].

### 2.5. Data Collection and Organization

Health data limitation is a problem that has faced GIS users in Turkey. To collect new data related to disease facts and to convert paper maps and data into digital format continues to be a problem. In many cases there are issues of confidentiality, national security, *etc*. which have prevented its use by health-related departments. One data problem that is particularly difficult to deal with cases address. While there may be some data available from the census, it is usually too old or not done frequently enough to be useful for epidemiologic research. The primary option is for health officers to conduct special surveys to determine the cases living locations. While this can result in accurate data, it cannot be used historically and takes time to collect. One way of approaching data problems is to set up a pilot program. A pilot program would have several benefits including: showing decision makers what is possible; working out problems on a small scale before launching an entire program nationwide; collecting data or converting if from analog format.

The data about cancer cases must include the patient’s age, sex, address, cancer type with topology, and diagnosis date. To investigate the locations of cancer cases in this study, Administrative Units and Roads spatial data sets are required for address geocoding. The Administrative Unit data sets include administrative boundaries as areas and allocation centers as points for districts and villages at the lowest administration level. To examine the relationship between geography and cancer cases, different types of spatial data sets should be produced and classified accordingly. Table 1 shows the database design and required data for this study.

The data related to cancer cases was obtained from the Trabzon Cancer Registry Center, which has been recording cancer cases diagnosed in Health Institutions of Trabzon since 2004. From that time the centre has recorded 1,216 cancer cases for the province. Cancer case data that did not have sufficient address information, and therefore, could not be identified, was eliminated. In this way, 1,150 cancer cases were examined with the data including the patient’s sex, age, address, and disease name/type, as shown in Table 1.

ArcGIS 9.x was used to combine the data and achieve spatial analysis. For all the spatial data sets, the datum is ITRF-96 (International Terrestrial Reference System) and the ellipsoid is GRS-80 (Geodetic Reference System-1980). The reference System is determined as ETRS89 Lambert Azimuthal Equal Area (ETRS-LAEA) because it has advantages for statistical analysis and presentation [35]. The Administration Unit Spatial Data Sets were collected from 1:25,000 and 1:100,000 scaled maps of the General Directorate of Rural Services, Trabzon Province Administration, and the sub institutions of Trabzon and was managed on a GIS environment. Population data for each Administrative Unit was collected from the Turkish Statistics Institute (TURKSTAT).

After matching the address related to each cancer case with the administrative unit data set, the location of cancer cases can be shown on the map that is produced. For each cancer case, an Administration Unit Code (IDBK) was determined and entered into the Cancer Registry dataset for geocoding at district/village level. This dataset was collected in dbase format as a spreadsheet. By using IDBK as a hierarchical unique value of each Administrative Unit, the number of cancer cases for each Administrative Unit was decided.

Elevation data sets were extracted from 1:25,000 scaled Digital Topography Maps and a Digital Elevation Model (DEM) was produced from the elevation data sets. Land Cover Spatial Data Sets were produced from LANDSAT 7 ETM+ (Enhance Tematic Mapper+) satellite images using RS supervised classification techniques. The spatial analysis function “Intersect” in ESRI ArcGIS was used to determine the relationship between cancer cases and topography and elevation.

### 2.6. Statistical Analysis

Incidence means the frequency with which a disease appears in a particular population or area. A cancer incidence rate is the number of new cancers of a specific site/type occurring in a specified population over a year, usually expressed as the number of cancers per 100,000 people at risk. The incidence rate for each administrative unit was calculated with Equation 1 below:

(1) Incidence rate = (New Cancer Cases/Population) ×100,000

According to the WHO, the number of cancer cases is estimated to be between a minimum 150 and maximum 300 for a population of 100,000 in developing countries [36] therefore, administrative units with a cancer incidence rate of more than 300 are considered to have a high cancer density level.

The Pearson chi-square (χ^2^) statistics test the independency between the sub categories of two variables of R*C (Row*Column). The importance of χ^2^p is determined, to compare critical values of χ^2^α, df and the freedom degree is calculated using df = (R – 1)(C – 1) [31]. SPSS ® version 10.0 was used for this statistical analysis.

## 3. Results and Discussion

### 3.1. Mapping of the Distribution of Cancer Cases

**Figure 2.**
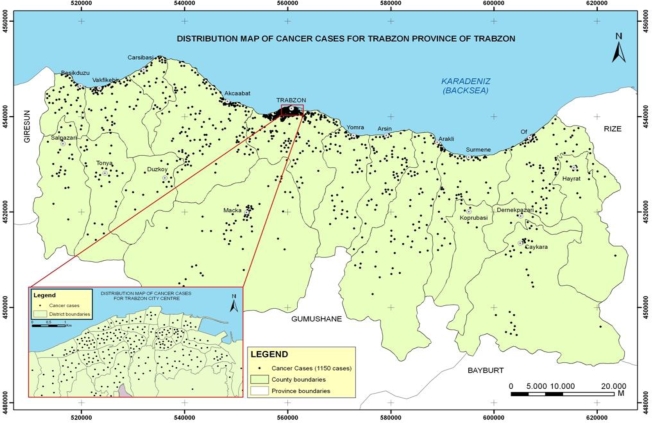
Distribution map of cancer cases.

After determining the location of cancer cases spatially, the distribution of cancer cases was examined using the Administrative Unit Spatial Data Set. The cases were classified in five common cancer types for Trabzon City. At the district and village level, the Distribution Map of Cancer Cases shown in Figure 2 was produced to visually present the frequency of cancer cases and types.

As seen on the map in Figure 2, more heavily populated administration units have more cancer cases, distributed in city and county centers, coastal residential areas, and across valleys. Investigating the 1,150 cancer cases, it was determined that the five common cancer types were lung (19.1%), skin (12.3%), breast (9.9%), stomach (9.5%), and bladder (6.8%), which is in keeping with the common cancers globally. Table 2 shows the percentage of different cancer types for men and women most notably showing that lung cancer is more prevalent in men and breast cancer more common in women.

### 3.2. The Cancer Incidence Map

**Figure 3.**
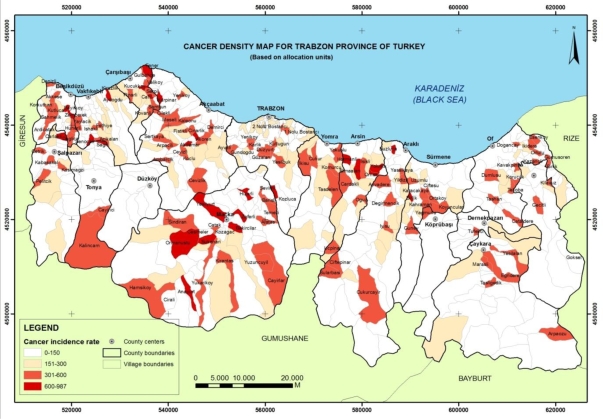
Cancer incidence map of Trabzon province in Turkey.

To analyze the distribution of cancer cases and cancer density on the map, the incidence rates of the administrative units were used as a comparison factor for the statistical research. After the number of cancer cases for each administrative unit was calculated, the incidence rate for each administration unit was determined using the population in the year 2004. The Cancer Density Map in Figure 3 was produced showing the incidence rates of the administrative units with classified groups at district and village level (see Table 3).

Administrative units with incidence rates of more than 300 are regions at risk in terms of cancer density are shaded in dark red on the map shown in Figure 3. It was determined that 138 administrative units, comprising 22% of all the administrative units, have a high incidence rate of cancer. The calculated incidence rate of Trabzon province was determined to be 118, thus confirm that overall the province has a lower incidence of cancer than that estimated by WHO for developing countries.

### 3.3. Relationship between the Cancer Cases and Land Cover

**Figure 4.**
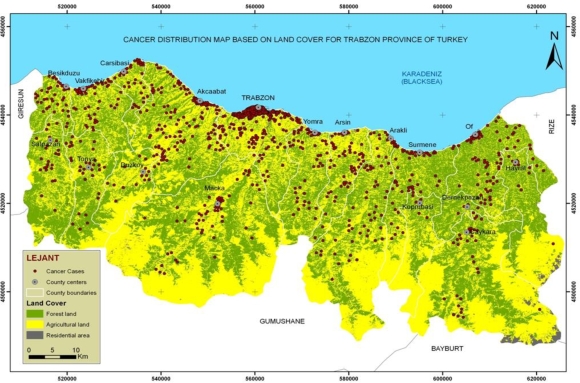
The distribution map of cancer cases based on land cover.

The distribution map of cancer cases based on land cover was produced to examine whether there is a relationship between land cover and cancer types. After producing the Trabzon Province Land Cover data sets like forest land, agriculture land and residential areas with supervised classification method as shown in Figure 4, the number of cancer cases in each land cover class was determined with the spatial analysis function “Intersect” as shown in Table 4.

Forty five cancer types and five land cover classes were determined in this study. To enable the statistical analysis, tea and hazelnut were combined with the Agriculture Land Cover Class. The Cancer Types were reduced to eight common cancer types taking related cancer types into consideration, as shown in Table 5. The number of cancer cases of each cancer type within each land cover class was calculated with a database query. To test whether there is a statistical relationship between land cover and cancer type, The Pearson chi square test was used and showed that there was a relationship between cancer types and land cover, χ^2^ = 24.391, df = 14, p = 0.041. Supposing the H_o_ (null) hypothesis to be true, count, the expected count, percentage, and adjusted residual were presented on the crosstabs of Table 5 for 1,150 cancer cases.

On examining the percentage of cancer cases by land cover classes as shown on Table 5, 47.8% corresponded to agriculture, 33.8% to forestry, and 18.3% to residential areas. On examining the percentage of the types of cancer cases, 22.3% of lung/bronchus/larynx/throat, 12.3% of skin, 9.9% of breast, 13.8% stomach, 6.8% of bladder, 4.9% of prostate, 4.8% of thyroid, and 25.1% of other cancer cases overlapped with classified land cover types, thus, making the supposition that the Ho hypothesis is false, showing that there is relationship between cancer types and land cover. Breast cancer occurred more in residential areas than other cancer types. Skin and thyroid cancer occurred more in forestry areas than the other cancer types. In addition, skin cancer cases in forestry areas have frequency values of, count = 61 and expected count = 48. 43% of skin cancer cases were diagnosed in forestry areas and these account for 5.3% of all cancer cases.

### 3.4. Relationship between the Distribution of Cancer Cases and Elevation

**Figure 5.**
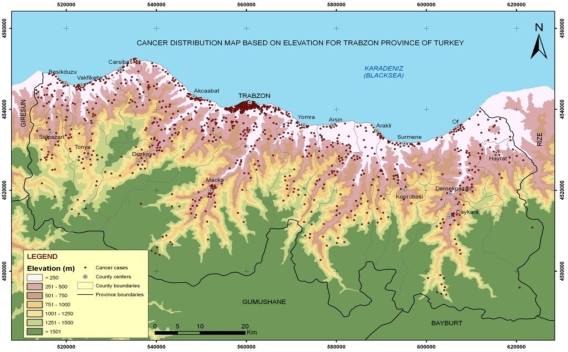
The distribution map of cancer cases based on elevation.

The distribution map of cancer cases based on elevation was produced to examine whether there is a relationship between elevation and cancer types. After producing a Digital Elevation Model (DEM) with elevation classes at 250 m intervals, as shown in Figure 5, the number of cancer cases of each cancer type in each elevation class was determined with the spatial analysis “Intersect” and database query as shown in Table 6.

The Table 6 has five elevation classes and eight cancer types. To test whether there is a statistical relationship between elevation and cancer type, The Pearson chi square test was used and indicated that there was a relationship between cancer types and elevation, χ^2^ = 46.466, df = 28, p = 0.016. If it is supposed that the Ho (null) hypothesis is true, count, expected count, percentage, and adjusted residual are presented in the crosstabs of Table 6 for 1,150 cancer cases.

Examining the percentage of cancer cases in the elevation classes as shown on Table 6, it was found that 54.2 % of all cancer types occurred at 0–250 m, 20.7% at 251–500 m, 11.1% at 501–750 m, 6.6% at 751–1,000 m, and 7.4% at an elevation of more than 1,000 m. If the Ho hypothesis is false, then there is relationship between cancer types and elevation.

In this case, there is a relationship between the 0–250 m elevation class and skin, breast and thyroid cancer types. In addition, breast cancer cases in the 0–250 m elevation have frequency values, count = 77 and expected count = 62. 67.5% of the Breast Cancer Cases occurred within the 0–250 m elevation and these are 6.7% of all cancer cases.

## 4. Conclusions

Today, GIS is not only a system employed for making disease maps but also leads to better understanding of the causative relationships between the environment and human health. Specifically, consideration of the relationship(s) between cancer cases and environmental factors is important in order to better manage cancer combating strategies and determining the causes of cancer. Therefore, creating geo-referenced maps is necessary to obtain valuable information about cancer cases with respect to frequency in spatial features. The great potential of GIS for health care management is just now beginning to be realized. Data integration and spatial visualization is now highly achievable with GIS. GIS can quickly make maps, and that maps are much easier to understand than tables. Because many do not understand what GIS does and what it could do, getting financial support continues to be a problem. This was a problem identified in the early days of GIS and it remains a problem today.

In this research, GIS applications were carried out in order to analyze the distribution of cancer types geographically. To determine cancer density statistics, the cancer incidence rates of the administrative units were calculated according to local census data then a cancer density map was produced. The ecological data such as land cover and elevation were combined and compared with the locations of cancer cases produced by address geocoding. The administrative units having a high incidence rate were generated combining both GIS and the Pearson chi-square (χ^2^) statistics analysis to locate areas where greater number of cancer cases occurred.

It is determined that the Trabzon province of Turkey has the number of cancer cases as anticipated by the WHO data, but only 23% of the villages and districts have more than the expected number of cancer cases. A relationship between breast cancer cases, land cover class and elevation class, was determined from statistical examinations. This showed that breast cancer cases commonly occurred in residential areas that are generally situated on the coast and along valleys and within the low elevation class. On the other hand, the numbers of skin and thyroid cancer cases within low elevation class were less than expected. In terms of land cover, the forestry areas, situated in high elevation classes were where more skin and thyroid cancer cases occurred.

In conclusion, the GIS and related spatial analysis methods provide a set of tools to describe and understanding the changing spatial organization of health care, to examine its relationship to health outcomes and access, and to explore how the delivery of health care can be improved. Epidemiologists, for example, have traditionally used maps when analyzing associations between location, environment, and disease. GIS is particularly well suited for studying these associations because of its spatial analysis and display capabilities.

## Figures and Tables

**Table 1.** Required spatial data themes.

**Data Theme**	**Data Sets**	**Data Set Name**	**Display**	**Attribute Name / Explanation**	
**Administrative Unit**	County	ILCE	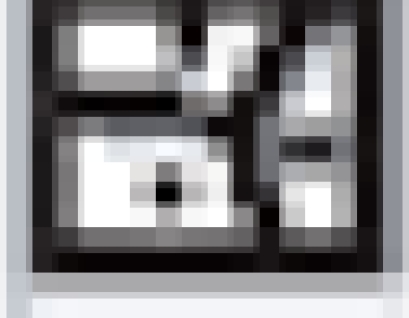 Polygon (Area)	IDBK ISIM …	Administration Unit Code Administration Unit Name
	
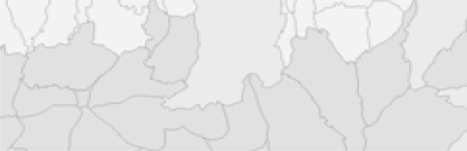	District/Village	MAKO	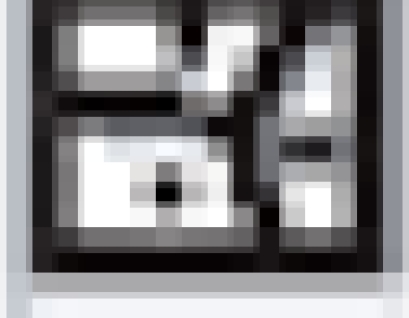 Polygon (Area)	IDBK ISIM IDBT NUFS …	Administration Unit Code Administration Unit Name Administration Unit Type Population

**Transportation** 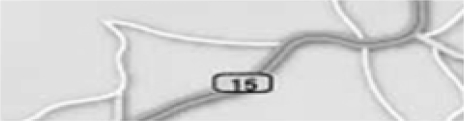	Road	YOLH	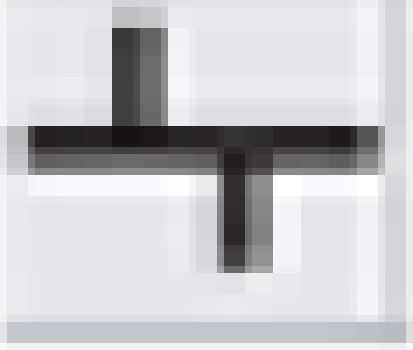 Line	ISIM YOTP BADK SADK …	Road Name Road Type Beginning Address Code Finishing Address Code

**Topography** 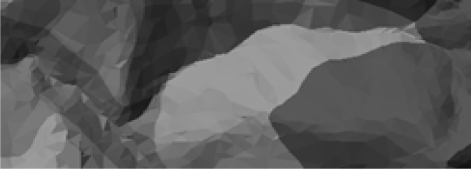	Elevation	YUKS	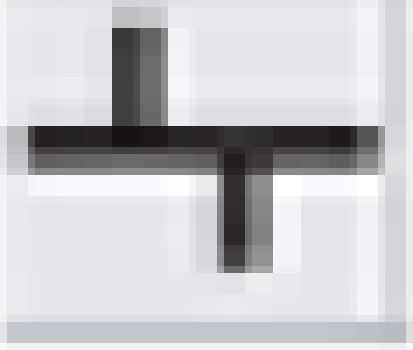 Line	YUKS …	Elavation
	Digital Elevation Model *Produced from Elavation*	SAYM	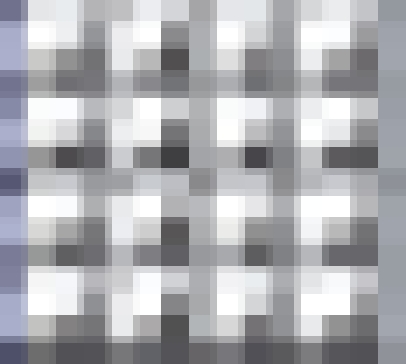 Raster	YUGR …	Elevation Classification

**Land Cover** 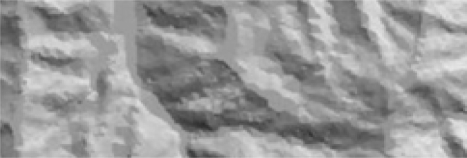	Satellite image		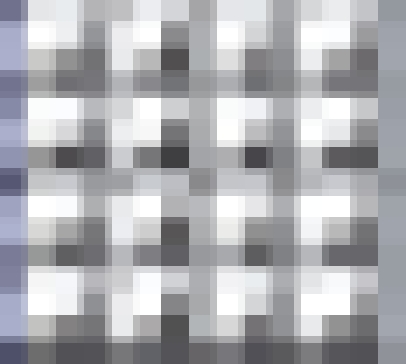 Raster	Landsat 7	ETM+ image, 2003
	Land Cover *Produced from Landsat7 ETM+ image*	AROR	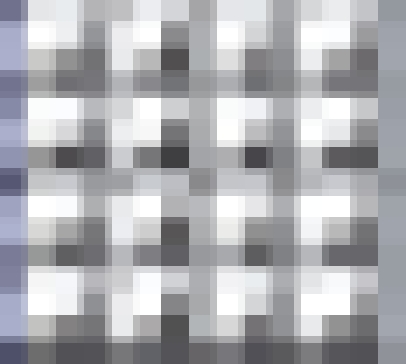 Raster	ARKS …	Land Cover Classification

**Health** 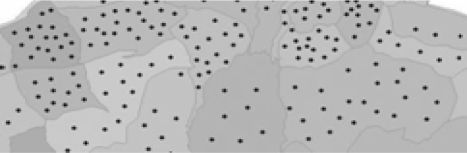	Cancer Registry	KAKA	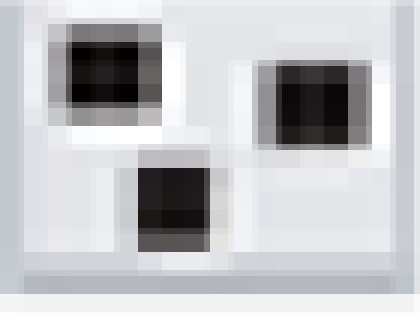 Point	SEX AGE ADRS IDBK TOPO TOPL DATE	Patient’s sex Patient’s age Patient’s address Admin. Unit of Address Disease Name Disease Type Diagnosis Date

**Table 2.** The most common cancer types with respect to sex in Trabzon (out of population 2004).

**Cancer Types**	**Man (%)**	**Women (%)**	**Total (%)**
Lung	28.5	5.2	19.1
Skin	11.1	14.3	12.3
Breast	0.3	24.2	9.9
Stomach	9.3	9.7	9.5
Bladder	10.5	1.3	6.8
Prostate	8.2	-	4.9
Thyroid	1.2	10.2	4.8
Other	30.9	35.1	32.7

**Table 3.** The number of administrative units on groups of incidence rates.

**Group of Incidence Rates**	**Number of Administrative Units**	**Percentage**
0	232	39
1–150	124	21
151–300	109	18
301–600	105	18
601–987	26	4

**Total**	**596**	**100%**

**Table 4.** The number of cancer cases within each land cover class.

**Land Cover Class**	**The number of Cancer Cases**	**Percentage**
Forestry	389	34
Agricultural area	299	26
Hazelnut	243	21
Residential area	211	18
Tea	8	1

**Total**	**1,150**	**100%**

**Table 5.** Crosstab presenting the relationship between land cover and cancer types.

**LAND COVER**		**CANCER TYPES**								
		**Lung/Bronchial &Larynx/Throat**	**Skin**	**Breast**	**Stomach & Colon/Rectum**	**Bladder**	**Prostate**	**Thyroid**	**Other Types**	**Total**
**Agricultural area**	Count									
	Expected count	125	62	52	85	34	27	23	142	550
	% of Total	122.9	67.9	54.5	76	37.3	26.8	26.3	138.2	550
	Adj.	10.9%	5.4%	4.5%	7.4%	3%	2.3%	2%	12.3%	47.8%
	Residual	0.3	−1.1	−0.5	1.5	−0.8	0.1	−0.9	0.5	

**Residential area**	Count									
	Expected count	55	19	29	25	12	9	5	57	211
	% of Total	47.2	26.1	20.9	29.2	14.3	10.3	10.1	53	211
	Adj.	4.8%	1.7%	2.5%	2.2%	1%	0.08%	0.4%	5%	18.3%
	Residual	1.4	−1.6	2.1	−0.9	−0.7	−0.5	−1.8	0.7	

**Forestry**	Count									
	Expected count	77	61	33	49	32	20	27	90	389
	% of Total	86.9	48	38.6	53.8	26.4	18.9	18.6	97.8	389
	Adj.	6.7%	5.3%	2.9%	4.3%	2.8%	1.7%	2.3%	7.8%	33.8%
	Residual	−1.5	2.5	−1.2	−0.9	1.4	0.3	2.5	−1.1	

**Total**	Count	257	142	114	159	78	56	55	289	1150
	% of Total	22.3%	12.3%	9.9%	13.8%	6.8%	4.9%	4.8%	25.1%	100%

**Table 6.** Crosstab presenting the relationship between elevation and cancer types.

**ELEVATION (Meter)**		**CANCER TYPES**								
		**Lung/Bronchus&Larynx/Throat**	**Skin**	**Breast**	**Stomach & Colon/Rectum**	**Bladder**	**Prostate**	**Thyroid**	**Other Types**	**Total**
**0–250**	Count	145	66	77	83	42	29	21	160	623
	Expected count	139.2	76.9	61.8	86.1	42.3	30.3	29.8	156.6	623
	% of Total	2.6%	5.7%	6.7%	7.2%	3.7%	2.5%	1.8%	13.9%	54.2%
	Adj. Residual	0.8	−2.0	3.0	−0.5	−0.1	−0.4	−2.4	0.5	

**251–500**	Count	53	37	23	29	15	9	17	55	238
	Expected count	53.2	29.4	23.6	32.9	16.1	11.6	11.4	59.8	238
	% of Total	4.6%	3.2%	2%	2.5%	1.3%	0.8%	1.5%	4.8%	20.7%
	Adj. Residual	0	1.7	−0.1	−0.8	−0.3	−0.9	1.9	−0.8	

**501–750**	Count	24	17	6	26	12	6	5	32	128
	Expected count	28.6	15.8	12.7	17.7	8.7	6.2	6.1	32.2	128
	% of Total	2.1%	1.5%	0.5%	2.3%	1%	0.5%	0.4%	2.8%	11.1%
	Adj. Residual	−1.0	0.3	−2.1	2.3	1.2	−0.1	−0.5	0	

**751–1000**	Count	14	12	5	8	3	10	5	19	76
	Expected count	17	9.4	7.5	10.5	5.2	3.7	3.6	19.1	76
	% of Total	1.2%	1%	0.4%	0.7%	0.3%	0.9%	0.4%	1.7%	6.6%
	Adj. Residual	v0.9	0.9	−1.0	−0.9	−1.0	3.5	0.8	0	

**>1000**	Count	21	10	3	13	6	2	7	23	85
	Expected count	19	10.5	8.4	11.8	5.8	4.1	4.1	21.4	85
	% of Total	1.8%	0.9%	0.3%	1.1%	0.5%	0.2%	0.6%	2%	7.4%
	Adj. Residual	0.5	−0.2	−2.0	0.4	0.1	−1.1	1.6	0.4	

**Total**	Count	257	142	114	159	78	56	55	289	1,150
	% of Total	22.3%	12.3%	9.9%	13.8%	6.8%	4.9%	4.8%	25.1%	100%
